# A balanced iterative random forest for gene selection from microarray data

**DOI:** 10.1186/1471-2105-14-261

**Published:** 2013-08-27

**Authors:** Ali Anaissi, Paul J Kennedy, Madhu Goyal, Daniel R Catchpoole

**Affiliations:** 1 Centre for Quantum Computation & Intelligent Systems (QCIS), Faculty of Engineering and Information Technology (FEIT), University of Technology, Sydney (UTS), Broadway New South Wales 2007, Australia; 2 The Tumour Bank, Children’s Cancer Research Unit, The Children’s Hospital at Westmead, Locked Bag 4001, Westmead New South Wales 2145, Australia

## Abstract

**Background:**

The wealth of gene expression values being generated by high throughput microarray technologies leads to complex high dimensional datasets. Moreover, many cohorts have the problem of imbalanced classes where the number of patients belonging to each class is not the same. With this kind of dataset, biologists need to identify a small number of informative genes that can be used as biomarkers for a disease.

**Results:**

This paper introduces a Balanced Iterative Random Forest (BIRF) algorithm to select the most relevant genes for a disease from imbalanced high-throughput gene expression microarray data. Balanced iterative random forest is applied on four cancer microarray datasets: a childhood leukaemia dataset, which represents the main target of this paper, collected from The Children’s Hospital at Westmead, NCI 60, a Colon dataset and a Lung cancer dataset. The results obtained by BIRF are compared to those of Support Vector Machine-Recursive Feature Elimination (SVM-RFE), Multi-class SVM-RFE (MSVM-RFE), Random Forest (RF) and Naive Bayes (NB) classifiers. The results of the BIRF approach outperform these state-of-the-art methods, especially in the case of imbalanced datasets. Experiments on the childhood leukaemia dataset show that a 7% ∼ 12% better accuracy is achieved by BIRF over MSVM-RFE with the ability to predict patients in the minor class. The informative biomarkers selected by the BIRF algorithm were validated by repeating training experiments three times to see whether they are globally informative, or just selected by chance. The results show that 64% of the top genes consistently appear in the three lists, and the top 20 genes remain near the top in the other three lists.

**Conclusion:**

The designed BIRF algorithm is an appropriate choice to select genes from imbalanced high-throughput gene expression microarray data. BIRF outperforms the state-of-the-art methods, especially the ability to handle the class-imbalanced data. Moreover, the analysis of the selected genes also provides a way to distinguish between the predictive genes and those that only appear to be predictive.

## Background

The huge number of gene expression values generated by microarray technology leads to very complex datasets, and many cohorts have the imbalanced classes problem (e.g. 80% alive vs. 20% deceased). These complexities raise the challenge of how to identify the biomarkers that are strongly associated with the disease and that can be used to distinguish classes of patients. Hence, feature selection is a critical technique in the field of bioinformatics [1] and it has been used in various domains for large and complex data, such as gene expression datasets.

Gene expression datasets are typically noisy and often consist of a limited number of observations (hundreds) relative to the large number of gene expression values (thousands of genes). In practical applications, datasets often exist in an unbalanced form. That is, at least one of the classes constitutes only a small minority of the data. For example, the following well-known and publicly available microarray datasets are imbalanced: malignant pleural mesothelioma (MPM) and lung adenocarcinoma (ADCA) gene expression dataset with a 17% class imbalanced (31 MPM versus 150 lung ADCA); acute lymphoblastic leukaemia (ALL) and acute myeloblastic leukaemia (AML) dataset with a 32% class imbalanced (23 samples of AML versus 49 samples of ALL). For problems such as these, the practical classification interest usually leans towards correct classification of the minor class. Generally, most of the classifiers used to select features suffer from the imbalanced classes and many have poor performance because they are biased to the large samples and pay less attention to the rare class. Consequently, unsatisfactory classification performance results and most of the rare class features are not recognized. These characteristics result in difficulties in working with standard machine learning techniques, which must be modified to deal with the complexities of gene expression data and to build an effective feature selection algorithm. These characteristics also adversely affect the analysis of microarray datasets that have received significant attention in the field of cancer diagnosis and treatment.

Acute Lymphoblastic Leukaemia (ALL) is the most common childhood malignancy [2]. It is a type of cancer that affects the blood and bone marrow. The causes of ALL are still unknown, but are thought to most likely result from mutations of genes [3]. Nowadays, ALL is diagnosed by a full blood count and a bone marrow biopsy. Based on these examinations, an ALL patient’s risk of relapse and appropriate treatment are identified. Most children achieve an initial remission, yet approximately 20% of children with ALL suffer a relapse [4]. This relapse problem, where the cancer recurs, is considered as one of the major obstacles to curing ALL patients. One reason for relapse is incorrect therapy due to mis-classification of risk factors of ALL patients [4]. Consequently, accurate risk assessment of patients is crucial for successful treatment.

With microarray technology, it is becoming more feasible to look at the problem from a genetic point of view and to perform genetic-based risk assessment for each patient. However, too many features or genes in a dataset adversely affect similarity measurement and classification performance, because many of these genes are irrelevant to specific traits of interest [5]. Consequently, biologists need to identify a small number of informative genes that can be used as biomarkers for the disease in order to understand gene expression in cells and to facilitate diagnosis and treatment of patients. To achieve this, a real childhood leukaemia gene expression dataset collected from The Children’s Hospital at Westmead is provided for this project that aims to identify biomarkers that are strongly associated with the risk of relapse of patients with the eventual aim of supporting clinicians and biologists in diagnosis and treatment of ALL patients. The dataset is composed of 110 patients and each patient has more than twenty two thousand gene expression values. Patients are classified into three categories based on the cancer’s risk type: standard, medium and high risk. The majority of 78 patients are classified as a medium risk, 21 patients are classified as a standard risk and the minority of 11 patients are classified as high risk. This imbalanced classes problem adversely affects the classification performance in the feature selection process, because it can result in a trivial classifier that classifies all patients as the majority class. Therefore, ignoring this critical data characteristic may result in very poor feature selection.

The random forest algorithm was developed by Breiman [6], and is known as one of the most robust classification algorithms developed to date. It is an ensemble classifier consisting of many decision trees. Many classification trees are grown during training. A training set is created for each tree by random sampling with replacement from the original dataset. During the construction of each tree, about one-third of the cases are left out of the selection and this becomes the out-of-bag cases that are used as a test set. The classification performance of the test set is evaluated based on the out-of-bag error rates.

Random forest has been used extensively in the biomedical domain [7,8] because it is well suited for microarray data. Features will not be deleted based on one decision or one tree, but many trees will decide and confirm elimination of features. Another positive characteristic of random forest is that it is applicable to very high dimensional data with a low number of observations, a large amount of noise and high correlated variables. Moreover, random forest is less prone to over-fitting and can handle the problem of imbalanced classes. All these characteristics make the random forest classifier an appropriate choice for gene expression datasets.

This paper addresses the problem of gene selection in the case of imbalanced datasets. Several authors have previously used random forest for gene selection but they haven’t addressed that complex problem (multi class-imbalanced data) and they did not take advantage of random forest in dealing with imbalanced classes. Diaz-Uriarte and Alvarez de Andres [7] explored the potential of random forest for attribute selection and proposed a method for gene selection using the out-of-bag error rates. The authors thoroughly examined the effects of changes in the parameters of random forest specifically, mtry, ntree and nodesize. However, the authors did not address the problem of imbalanced classes and how the parameters cutoff and sampsize can handle that problem. Archer and Kimes [8] performed a similar evaluation of the random forest classifier and achieved feature selection using variable importance measures obtained by random forest, but they did not address the problem of imbalanced classes. Moorthy et al [9] also use random forest for gene selection based on the out-of-bag-error rates. The only difference is that [9] aims to obtain the biggest subset of genes with the lowest error rates. They have performed experiments to see whether the classification performance of the larger subset of genes outperformed the smaller subset of genes. These experiments also have been performed in this paper, but with the consideration of the classification performance effects on the imbalanced classes. Overall, these proposed methods [7]*-*[9] might not be the appropriate choice for our purposes as we have to select genes from imbalanced data. This paper also considers the problem of over-fitting, which must be addressed in any machine learning algorithm that is dealing with datasets having a low number of samples compared to a very high number of attributes. Finally, and as in [7], the last issue addressed in this paper is the evaluation of the selected genes to determine whether they are stable and appear in multiple executions, or selected only once.

This paper proposes a feature selection method called Balanced Iterative Random Forest (BIRF) to select genes that are relevant to a specific trait of interest from gene expression datasets. This work is different to the previous approaches because it enhances the gene selection process of imbalanced data by tuning the parameters cutoff and sampsize of the random forest classifier.

## Methods

### Balanced iterative random forest for feature selection

This paper introduces a new method for feature selection based on random forest called Balanced Iterative Random Forest (BIRF). Balanced iterative random forest is an embedded feature selector that follows a backward elimination approach. The base learning algorithm is random forest, which is involved in the process of determining what features are removed at each step. The algorithm starts with the entire set of features in the dataset. At every iteration, the number of the attributes is reduced by removing those attributes that have zero importance value. After discarding those genes, a new random forest is built with the selected set of genes that yields the smallest out-of-bag (OOB) error rate.

This algorithm is mainly tested on the real childhood leukaemia gene expression dataset collected from The Children’s Hospital at Westmead. All specimens, as well as the associated comprehensive patient clinical data, used to generate the microarray dataset upon which we developed the BIRF algorithm, were made available to the chief investigators with the approval of and according to the guidelines established by the Children’s Hospital at Westmead’s Human Research Ethics Committee and Tumour Bank Committee and is compliant with the Declaration of Helsinki.

The R package randomForest is used in this paper. The two main parameters of random forest are mtry, the number of input variables randomly chosen at each split and ntree, the number of trees in the forest. These two parameters are set to their default values $(\text{ntree}=500;\;\text{mtry}=\sqrt{d}$, where *d* is the number of features). Two other parameters are very important in this algorithm due to the problem of imbalanced classes and ignoring them may result in poor feature selection. The two parameters are cutoff, a vector weight for each class, and sampsize, the number of cases to be drawn to grow each tree. These two parameters are carefully tuned in order to achieve a successful feature selection process that able to recognize features in the minority classes and not ignoring them.

Similar to standard classifiers, random forest also has the problem of learning from extremely imbalanced class datasets. However, random forest has the capacity to mitigate this problem, and two solutions are applied on the BIRF to alleviate it: balanced sample and cost sensitive learning. The balanced sample solution is based on the parameter sampsize, which aims to induce random forest to build trees from a balanced bootstrap sample, which is a bootstrap sample that is drawn from the minority class with the same number of samples from the majority class. In the case of imbalanced data, there is a high probability that random forest will build a tree from a bootstrap sample that contains only a few samples from the minority class, resulting in poor performance for predicting the minority class.

The second solution aims to apply a cost sensitive learning technique through the parameter cutoff in order to make random forest more suitable for learning extremely imbalanced data. Cost sensitive learning assigns a high cost for mis-classification of the minority class and minimisation of the cost of the major class. As random forest generates votes to classify the input case, cost weights are applied on those votes in order to make the calculation of the votes as proportion, rather than whole. This solution aims to balance the distribution of classes without altering the semantics of the dataset or by down-sampling or over-sampling the dataset.

#### Algorithm of balanced iterative random forest

A balanced iterative random forest algorithm is proposed to select the most relevant genes for the disease and can be used in the classification and prediction process. Due to the large size of gene expression datasets, and in order to have a fast feature selection process, it was not practical to run BIRF algorithm on all genes of the dataset because it takes too long. Consequently, we split the data, by the number of genes, randomly into different datasets only in the first iteration of the algorithm. This splitting of the dataset is optionally in the BIRF algorithm. By splitting the dataset, BIRF will run fast, but random forest may lose some global correlation in the first iteration However, it will be able to include it in the rest of the algorithm. Without splitting, the BIRF algorithm takes too long to run, but it is able to include the global correlation in all iterations.

The BIRF algorithm (including splitting the data) is run on each dataset to select the informative genes. The selected genes from each dataset are then combined to form a new gene expression dataset with fewer attributes. With this obtained dataset, BIRF then begins an iterative attribute elimination process and without losing the global correlation. It is presented below as Algorithm 1.

#### Validation of over-fitting

Over-fitting occurs in statistics and machine learning algorithms especially when these algorithms are dealing with complex datasets, such as gene expression datasets (many attributes relative to small number of samples) [5]. We also established in the Background that one of the characteristics of random forest is that it is less prone to over-fitting. Nevertheless, to further support the process of feature selection, additional experiments are performed to ensure that there is no over-fitting in the gene selection process. Early-stopping [10]*,*[11] is used here to avoid over-fitting by stopping the elimination of genes once over-fitting starts to happen. This is achieved by splitting the training set into a new training set and a validation set, which is used in the genes selection process to decide when to stop. In each iteration, after removing the irrelevant genes from the new training set, the same genes are eliminated from the validation set and classification performance is evaluated on the validation set (see Algorithm 1). Once the classification error rate of the validation dataset starts to increase after reaching a minimum value, it is assumed that the new training set is over-trained and that the algorithm should stop at this stage.

#### Validation of the selected genes

The decision about how many attributes to use during the feature selection process is critical and has two effects. Selecting too many attributes from the original dataset makes it difficult to analyse these genes in terms of their effect on the disease. On the other hand, in order to build a generalizable classifier or gene-based similarity measurement model, it is important to incorporate as much information as possible. Therefore, it is possible to make a principled decision by testing the effect of the selected number of attributes on the classification performance to know whether more genes provide new information or not.

Although the error rate of the validation dataset with the selected genes may reach a minimum value and provides a good classification performance, these selected genes may still require further exploration to determine whether they are globally informative or if they are just selected by chance and may be only predictive to that particular dataset. In order to support the gene selection process and to distinguish between predictive attributes and those that only appear to be predictive, this paper proposes a methodology to decide what genes best describe the original dataset. The methodology repeats experiments, training the BIRF algorithm several times and reduces the training dataset into several subsets (resultant attribute lists). The resultant attributes in each subset are then compared to see what attributes are selected in multiple executions, and which attributes are only selected once. The assumption is that the attributes that appear in multiple subsets are more informative than attributes that appear in a single subset. The subset that contains the most common attributes with the minimum error rates on the validation dataset is the one that best describes the original dataset.

## Results and discussion

Several experiments are performed on the balanced iterative random forest algorithm in order to demonstrate the validity of the proposed algorithm, to evaluate the algorithm on different datasets and to compare our achieved results to other algorithms by using the same datasets.

### Datasets

The experiments are performed on a childhood leukaemia gene expression dataset that has been collected from The Children’s Hospital at Westmead. This dataset is also available in the public domain and can be explored through the Oncogenomics Section of the Paediatric Oncology Branch at the National Cancer Institute NIH, USA ( http://pob.abcc.ncifcrf.gov/cgi-bin/JK). The dataset was normalized by the Distance Weighted Discrimination (DWD) algorithm [12]. The entire childhood leukaemia gene expression dataset is composed of 110 patients with expression values for 22,678 genes. However, stratified random sampling is applied on the gene expression dataset and it is divided into training and test datasets. The training dataset is composed of 70 patients who are classified as follows: 

• Standard risk (11 patients)

• Medium risk (53 patients)

• High risk (6 patients)

The test dataset is composed of 40 patients and they are classified as follows: 

• Standard risk (10 patients)

• Medium risk (25 patients)

• High risk (5 patients)

Three other publicly available microarray datasets: NCI 60, Colon cancer and Lung cancer datasets have been used in this paper for evaluation of BIRF. These datasets are characterized by a relatively small number of samples with a high dimensional space. For the two datasets (Colon and Lung), the same training and test data reported in the previous studies are used in these experiments, without changing the sample sizes, so that the obtained results can be objectively compared with earlier published results. However, a stratified random sampling is applied on the NCI 60 dataset and it is divided into training and test datasets. 

• NCI 60 dataset is a well-studied publicly available microarray benchmark collected by Ross et al [13] and is produced using Affymetrix HG-U133A chips. The data we used is the same as the data used in [7]. The dataset consists of 61 samples that are classified into eight categories. Each sample is measured over 5,244 gene expression values (see Table 1).

• Colon dataset is a publicly available microarray dataset that was obtained with an Affymetrix oligonucleotide microarray [14]. The Colon dataset contains 62 samples, with each sample containing the expression values for 2000 genes. Each sample indicates whether or not it came from a tumour biopsy. This dataset is used in many different research papers on feature selection of gene expression datasets [15-17]. The dataset is quite noisy but the real challenge is the shape of the data matrix where the dimensionality of the feature space is very high compared to the number of cases. It is important to avoid over-fitting in this dataset. Although the number of cases is very low, the dataset is split into two: a training dataset and a test dataset composed of 38 and 34 samples, respectively (see Table 1).

• Lung cancer dataset is also used in the experiments and it was generated with an Affymetrix oligonucleotide microarrays and normalized by z-score [18]. Each sample it indicates whether it came from a malignant pleural mesothelioma (MPM) or adenocarcinoma (ADCA). There are 181 tissue samples (31 MPM and 150 ADCA) that have already been broken into training and testing samples. The training dataset contains 32 of samples, 16 MPM and 16 ADCA. The remaining 149 samples are used for testing. Each sample is described by 12533 genes. Similar to the Colon dataset, the Lung cancer dataset is also noisy but with more samples and genes. These samples are broken into two datasets: a training dataset and test dataset composed of 32 and 149 samples, respectively (see Table 1).

**Table 1:** Microarray gene expression datasets

**Datasets**	**Number of classes**	**Number of features**	**Number of training samples**	**Number of testing samples**	**Profiles**
childhood leukaemia	3	22,678	70	40	Risk of relapse
NCI	8	5,244	45	16	8 phenotypes
Colon cancer	2	2,000	38	34	Cancer/Normal
Lung cancer	2	12,533	32	149	MDM/ADCA

### Experiments on childhood leukaemia dataset

Balanced Iterative Random Forest is validated with the childhood leukaemia gene expression dataset collected from The Children’s Hospital at Westmead. It is important to note that the main purpose of these experiments is to find a subset of genes most closely correlated with the leukaemia risk type distinction. Also, it is important to incorporate as much data as possible without including so much data that it may result in losing interesting separations between patients. The set of informative genes to be used in the prediction of risk type was chosen to be the 107 genes (see Additional file 1) selected at a lowest error rate 0.04. In order to validate the results obtained from this experiment, the test dataset is processed by random forest to view the classification performance of the selected genes. Table 2 shows the confusion matrix for classification of the test dataset and demonstrates that the classifier generalised reasonably well.

**Table 2:** A confusion matrix for the childhood leukaemia test dataset

	**Predicted high**	**Predicted medium**	**Predicted standard**
Actual High	3	1	1
Actual Medium	0	22	3
Actual Standard	0	2	8

#### Validation of Results in terms of Over-Fitting

**Figure 1:**
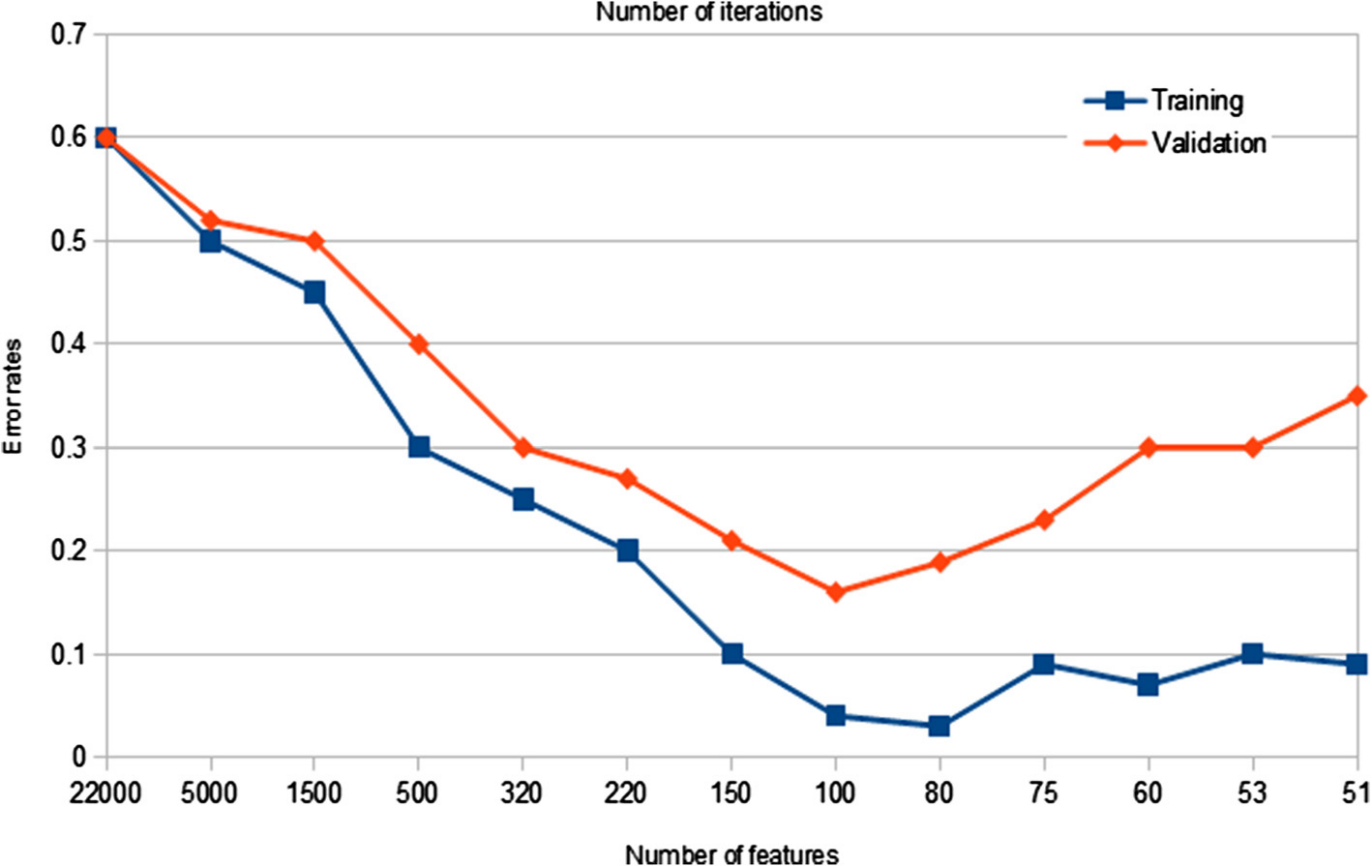
Comparison variations the error rates of the validation and training datasets during selecting the features from childhood leukaemia dataset.

It is important to be able to validate results and prove that they are not due to over-fitting of the training data. The Early-stopping is used here and iterative random forest is run again on the training dataset to select the relevant biomarkers. Simultaneously, the validation dataset is involved in this process to ensure that the training process does not over-train. The training process of the iterative random forest on the childhood leukaemia dataset is shown in Figure 1.

As can be seen from the graph and based on the training dataset, the out-of-bag error decreases as the number of irrelevant features and noise is eliminated at each iteration. After several iterations, the out-of-bag error becomes stable in a range between 0.1 and 0.04. With respect to the validation dataset, the error rates also decreases as the number of irrelevant features from the training dataset are eliminated at each iteration. The error rates of the validation dataset consistently decreases in the first eight iterations. After the eighth iteration, the error rates of the validation dataset increases again and becomes unstable for several iterations. The training stops at the eighth iteration when the lowest error rates are achieved for the validation dataset (0.16). It is important to note that there is no over-training of the dataset in the first eight iterations and that the number of features is greater than 100. After the ninth iteration, the error rates of the validation dataset starts to increase again after reaching the minimum.

#### Analysis of selected genes

To further evaluate the attribute-selection process, experiments with the balanced iterative random forest algorithm are repeated three times. The resultant attribute lists from each repetition are then compared to the attributes obtained from the initial experiment where 107 genes have been selected. The goal is to see whether the 107 selected attributes appear in the three resultant attribute lists, or not. It is interesting to note that 80% of the top 20 genes consistently appear in the three lists, and the top 20 genes remain near the top in the other three lists. Sixty four percent of the top 100 selected genes from each list are the same. This supports the fact that the top selected genes are globally predictive and have not been selected by chance. Moreover, it also indicates that the feature selection process was not over-trained.

Classification performance of the three resultant attribute lists are also compared to see whether the list that contains the most common attributes provides good separation between the patients. The error rates of the of the three lists are 0.28, 0.21 and 0.18, respectively. It can be clearly seen from this analysis that the dataset with the selected 107 genes (see Additional file 1) contains the most common attributes. It provides the minimum error rates (0.16) and is the best for describing the original dataset.

### Experiments on the three public microarray datasets

One of the most important aspects of any experiment is validating the algorithm. Validation is achieved by applying the proposed algorithm on three publicly available microarray datasets. If the algorithm performs well then the feature selection process has been completed correctly.

**Figure 2:**
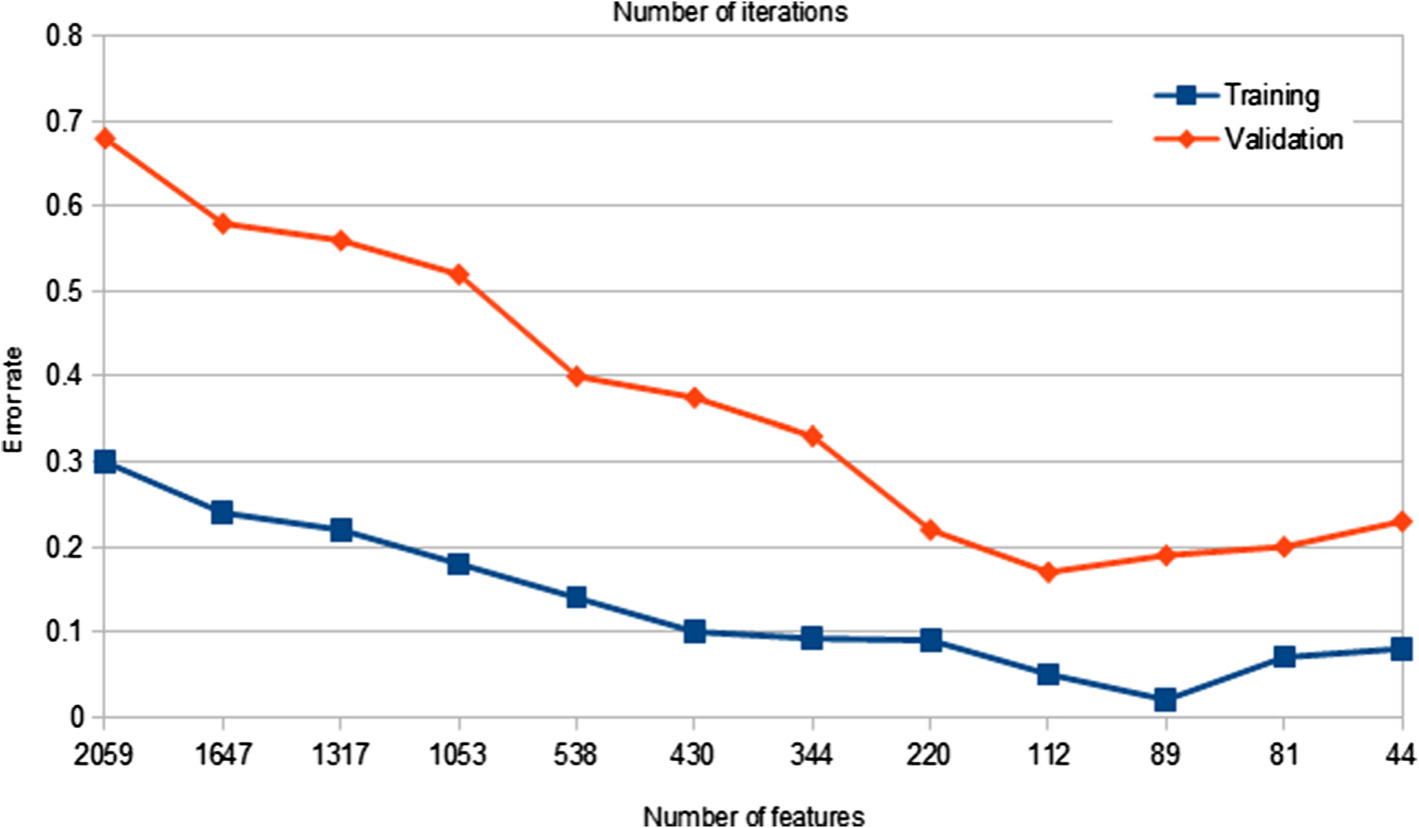
Comparison variations the error rates of the training and validation datasets during selecting the features from NCI60 dataset.

Balanced Iterative Random Forest is initially validated on the NCI 60 dataset. The same procedure, that is early stopping, is applied on the NCI 60 dataset in order to validate the results in terms of over-fitting. The training process of the iterative random forest on the NCI60 dataset is shown in Figure 2. The graph shows that the out-of-bag error rates of the training dataset decreases until they reach the minimum reduction of genes, which is realized at 89 genes with the lowest out-of-beg error rates 0.02. With respect to the validation set, the minimum error rates of 0.17 are achieved at 112 genes. The error rates are then increased again to reach 0.23 at 44 genes. The number of the selected genes from this process is 112 with a 0.17 error rates.

Balanced Iterative Random Forest is also validated on the Lung cancer dataset [19]. The minimal o error rates of zero is achieved at 57 features, which are selected as the most important features for classification. This result is also validated in order to ensure that the feature selection process has not over-fitted to that training dataset. With the selected 57 features, 97% accuracy have been achieved on the test dataset with only one patient is wrongly classified.

The same procedure is applied to the Colon dataset [14]. Nineteen features are selected as the most important features in classification with a minimal error rates of zero. An accuracy of 96% has been achieved for the test data where only one patient is wrongly classified. These results suggest that BIRF works well for several gene expression datasets.

### Comparison with other state-of-the-art algorithms

In the previous section, we performed experiments on two different public gene expression datasets that have been analysed by researchers using various gene selection methods. We compare the classification performance of the variable selection approaches used by the following two classifiers: 

• **Support Vector Machines (SVM)**: SVM are considered as one of the best performers for a number of classification tasks ranging from text to microarray data [20]. The goal of SVM is to find the optimal hyperplane that separates the classes. This hyperplane separates the classes into two categories. In case the target data has more than two categories, several approaches have been proposed, but the one used here is one-versus-one (OVO) SVM [21] as implemented in [20]. More introductions and description of SVM can be found in [22].

• **Naive Bayes (NB)**: NB is a simple probabilistic classifier based on the so-called Bayesian theorem. The goal of NB is to calculate the probability for a given case in order to assign it to a certain class. Naive Bayes assumes that the features constituting the case contribute independently for a given class. Naive Bayes is used for predicting miRNA genes [23], emotion recognition [24] and gene selection [25].

We report the results achieved by Support Vector Machine-Recursive Feature Elimination (SVM-RFE), Multiple SVM-RFE (MSVM-RFE), Random Forest (RF) based backward elimination procedure [7] and Naive Bayes (NB) as shown in Table 3. We have used the standard error rates, that is, subtracting the predicted samples from the actual samples and then dividing it by the actual samples. The error rates in BIRF are calculated using the independent sub-sample method. However, in [20], the authors calculate the error rates using Leave-one-out cross validation, and in [7], the authors use the bootstrap sample. The best performance on the Colon dataset is achieved at 96% obtained by BIRF. The accuracy of SVM-RFE, MSVM-RFE, RF and NB is 83.71% [20], 83.57% [20], 87% [7] and 87% [25], respectively. With respect to the Lung cancer dataset, 81% and 88% accuracies are reported (not shown in Table 3) using the bagging and boosting methods [26] where 97% is achieved by BIRF and standard random forest. The results were not available (NA) for RF and NB in the lung cancer dataset because no results were provided in the references [7,25].

**Table 3:** Accuracy results for Colon and Leukaemia datasets

**Datasets**	**Measurements**	**BIRF**	**SVM-RFE from **[20]	**MSVM-RFE from **[20]	**Random forest from **[7]	**NB from **[25]
Colon	Number of genes	19	7	3	15	2
	Accuracy	0.96	0.83	0.83	0.87	0.87
Lung	Number of genes	57	31	33	NA	NA
	Accuracy	0.97	0.96	0.96	NA	NA

### Comparison of BIRF, RF and MSVM-RFE on the childhood leukaemia dataset

We have compared the performance of BIRF to MSVM-RFE and RF gene selection methods to show the predictive performance of BIRF, particularly on the childhood leukaemia dataset. Multiple SVM-RFE is a widely used gene selection method that involves iteratively fitting SVM classification models by eliminating the genes with the low impact on classification in order to produce a small subset of genes that provides the best classification model. One-versus-one (OVO) SVM [21] is used in these experiments for a multi-class dataset. On the other hand, random forest based backward elimination procedure involves iteratively fitting the random forest model. At each iteration, genes with the smallest importance value are removed and a new random forest model is built with less number of genes and smallest out-of-bag error rates.

The childhood leukaemia dataset is used here to compare the gene selection performance of BIRF to MSVM-RFE. At each number of selected genes, SVM and random forest models are built on the training dataset with the selected genes using the leave-one-out method to compute the accuracy of classifiers. Table 4 shows the performance prediction of the two classifiers at different numbers of selected genes. As can be seen from the table, the accuracies of the two classifiers increased as the number of the selected genes decreased until the two classifiers achieved the maximum accuracies. At that stage, both classifiers’ accuracies decreased as the number of genes decreased. The highest accuracy of BIRF is achieved at 0.99. With that accuracy, all the patients in the two minor classes (High and Standard Risk) are predicted correctly, while 97.1% were predicted from the majority class (Medium Risk). The highest accuracy achieved by MSVM-RFE is 0.92. However, the accuracies of the High, Medium and Standard risk patients are 88.2%, 100% and 88.8%, respectively. From this comparison we can conclude that BIRF outperforms MSVM-RFE, especially in predicting the patients in the minor class.

**Table 4:** Comparison of BIRF and MSVM-RFE on childhood leukaemia dataset

**BIRF**		**MSVM-RFE**	
Number of Features	AUC	Number of Features	AUC
1027	0.54	1024	0.4
540	0.68	512	0.64
221	0.87	256	0.86
127	0.98	128	0.92
			H:88.2% M:100% S:88.8%
107	0.99	64	0.88
	H:100% M:97.1% S:100%		
98	0.92	32	0.79
85	0.95	16	0.69

The two built models (SVM and random forest) that provide better prediction are then used on the independent test data (i.e. childhood leukaemia test dataset) to assess the accuracies of the classifiers with the selected genes. The AUC (area under the ROC curve) of the random forest model built on the 107 BIRF-selected biomarkers is 0.874. However, the SVM model built on the 128 MSVM-RFE-selected biomarkers has an AUC of 0.751.

Random Forest (RF) based backward elimination procedure [7] is also applied to the childhood leukaemia gene expression dataset. This method completely failed to predict patients in the minority classes without handling the problem of imbalanced classes. This result suggests that standard random forest has to be modified in order to consider the problem of a cohort existing in an imbalanced form.

## Conclusion

This paper proposes a method called balanced iterative random forest to select features from imbalanced gene expression datasets. Feature selection as one of the most important processes in the field of microarray data has been considered carefully in this paper. This paper shows that the feature selection process is undertaken in an intelligent way, especially the way in which the imbalanced classes and over-fitting problems are handled, and when the selected genes are evaluated by reducing the dataset into several subsets of varying sizes to see whether the selected genes are stable and appear in the multiple subsets. It is unrealistic to assume that the attribute-selection algorithm, in this case the balanced iterative random forest algorithm, will be able to pinpoint what attributes can describe the risk type of the patient and identify all of the biologically significant attributes with such a large and complex dataset. Nevertheless, the attribute selection process is undertaken carefully by validating the results, and it produces a small subset containing the most informative genes. This result was validated and supported through two different experiments: over-fitting validation and analysis of the selected genes. The experiments demonstrated that the classifier did not over-fit the training dataset. Also, the analysis of attributes to distinguish between predictive attributes and those that only appear to be predictive (over-fitted attributes) showed that most of these attributes appeared in multiple repeats of the algorithm runs. However, BIRF algorithm has a limitations that is Random Forest will not be able to get global correlation due to the splitting of the dataset but this is optional and can be avoided if you don’t want to run BIRF fast or you have a powerful machine. Another limitation is tuning the parameter cutoff which is responsible handling the imbalanced classes problem. Balanced Iterative Random Forest is also applied to three other microarray datasets: NCI 60, Colon cancer and Lung cancer datasets. Overall, BIRF resulted in classifiers comparable or superior in accuracy to SVM-RFE, MSVM-RFE, RF and Naive Bayes on the Colon and Lung datasets.

## Competing interests

The authors declare that they have no competing interests.

## Authors’ contributions

AA and PJK conceived the algorithm and designed the experiments. MG proposed comparing the algorithm to the other state-of-art methods. AA performed experiments and analysed the data. DRC worked on the biological analysis of the selected genes. All authors read and approved the paper.

## Supplementary Material

Additional file 1 This additional file shows the information about the selected genes which validated through gene ontology. Click here for file
