# Supplementary material for: A balanced iterative random forest for gene selection from microarray data
Source: BMC Bioinformatics. 2013 Aug 27;14:261. doi: 10.1186/1471-2105-14-261 (PMC3766035; doi:10.1186/1471-2105-14-261)
Supplement: Additional file 1 — This additional file shows the information about the selected genes which validated through gene ontology. [file 1471-2105-14-261-S1.pdf]

210249 s_at	NCOA1	Nuclear receptor coactivator 1
217728_at	S100A6	S100 calcium binding protein A6
207426_s_at	TNFSF4	Tumor necrosis factor superfamily, member 4
213056_at	FRMD4B	FERM domain containing 4B
206995_x_at	SCARF1	Scavenger receptor class F, member 1
209995_s_at	TCL1A	T-cell leukemia/lymphoma 1A
209808_x_at	ING1	Inhibitor of growth family, member 1
200660_at	S100A11	S100 calcium binding protein
208438_s_at	FGR	Gardner-Rasheed feline sarcoma viral oncogene
222146_s_at	TCF4	Transcription factor 4
217939_s_at	AFTPH	Aftiphilin
218281_at	MRPL48	Mitochondrial ribosomal protein
39318_at	TCL1A	T-cell leukemia/lymphoma 1A
212587_s_at	PTPRC	Protein tyrosine phosphatase, receptor type, C
207416_s_at	NFATC3	Nuclear factor of activated T-cells calcineurin-dependent 3
218847_at	IGF2BP2	Insulin-like growth factor 2
209107_x_at	NCOA1	Nuclear receptor coactivator 1
212386_at	TCF4	Transcription factor 4
212588_at	PTPRC	Protein tyrosine phosphatase, receptor type, C
210555_s_at	NFATC3	Nuclear factor of activated T-cells calcineurin-dependent 3
217542_at	MGC5370	Hypothetical protein MGC5370
201461_s_at	MGC5370	Hypothetical protein MGC5370
203753_at	TCF4	Transcription factor 4
203434_s_at	MME	Membrane metallo-endopeptidase
213891_s_at	TCF4	Transcription factor 4
208720_s_at	RBM39	RNA binding motif protein 39
203435_s_at	MME	Membrane metallo-endopeptidase
217979_at	TSPAN13	Tetraspanin 13
208894_at	HLADRA	Major histocompatibility complex, class II, DR alpha
210982_s_at	HLADRA	Major histocompatibility complex, class II, DR alpha
204689_at	HHEX	Hematopoietically expressed homeobox
207805_s_at	PSMD9	Proteasome 26S subunit
209644_x_at	CDKN2A	Cyclin-dependent kinase inhibitor 2A
221569_at	AHI1	Abelson helper integration site 1
220068_at	VPREB3	Pre-B lymphocyte gene 3
200026_at	RPL34	Ribosomal protein L34
205548_s_at	BTG3	BTG family, member 3

214003_x_at	RPS20	Ribosomal protein S20
216307_at	DGKB	diacylglycerol kinase, beta 90kDa
219451_at	MSRB2	methionine sulfoxide reductase B2
217413_s_at	TNXB	tenascin XB
221952_x_at	TRMT5	tRNA methyltransferase 5 homolog (S. cerevisiae)
206287_s_at	ITIH4	inter-alpha-trypsin inhibitor heavy chain family, member 4
202236_s_at	SLC16A1	solute carrier family 16, member 1 (monocarboxylic acid transporter 1)
212423_at	ZCCHC24	Zinc finger, CCHC domain containing 24
200032_s_at	RPL9	Ribosomal protein L9
202695_s_at	STK17A	Serine/threonine kinase 17a
205726_at	DIAPH2	Diaphanous homolog 2 (Drosophila)
204075_s_at	KIAA0562	Uncharacterized protein
217820_s_at	ENAH	Enabled homolog (Drosophila)
200062_s_at	RPL30	Ribosomal protein L30
203577_at	GTF2H4	General transcription factor IIH, polypeptide 4
204218_at	C11orf51	Chromosome 11 open reading frame 51
203233_at	IL4R	Interleukin 4 receptor
203616_at	POLB	Polymerase (DNA directed), beta
212810_s_at	SLC1A4	Solute carrier family 1
209152_s_at	TCF3	Transcription factor 3
221543_s_at	ERLIN2	ER lipid raft associated 2
212324_s_at	VPS13D	Vacuolar protein sorting 13
201094_at	RPS29	Ribosomal protein S29
208690_s_at	PDLIM1	PDZ and LIM domain 1
206752_s_at	DFFB	DNA fragmentation factor
215000_s_at	FEZ2	Fasciculation and elongation protein zeta 2
203688_at	PKD2	Polycystic kidney disease 2
205786_s_at	ITGAM	Integrin, alpha M
217168_s_at	HERPUD1	Homocysteine and ER stress-inducible, ubiquitin-like domain member 1
203414_at	MMD	Monocyte to macrophage differentiation
218380_at	NLRP1	NLR family, pyrin domain containing 1
211991_s_at	HLADPA1	Major histocompatibility complex
210776_x_at	EST63624	Jurkat T-cells V Homo sapiens cDNA 5- end, mRNA sequence
200602_at	APP	Amyloid beta (A4) precursor protein
